# Thrombospondin‐1 mediates Drp‐1 signaling following ischemia reperfusion in the aging heart

**DOI:** 10.1096/fba.2019-00090

**Published:** 2020-03-20

**Authors:** Natia Q. Kelm, Jason E. Beare, Gregory J. Weber, Amanda J. LeBlanc

**Affiliations:** ^1^ Cardiovascular Innovation Institute University of Louisville Louisville KY USA; ^2^ Kentucky Spinal Cord Injury Research Center University of Louisville Louisville KY USA; ^3^ Department of Physiology University of Louisville Louisville KY USA

**Keywords:** aging, dynamin-related protein-1, ischemia reperfusion, reactive oxygen species, thrombospondin 1

## Abstract

**Background:**

Ischemia reperfusion (IR) injury leads to activation of dynamin‐related protein (Drp‐1), causing mitochondrial fission and generation of reactive oxygen species (ROS), but the molecular mechanisms that activate Drp‐1 are not known. The purpose of this study was to establish a link between Thbs‐1 and fission protein (Drp‐1) through Pgc‐1α following IR in advancing age.

**Methods:**

Female Fischer‐344 rats were divided into four groups: Young Control, Young + IR, Old Control, and Old + IR. Heart function and coronary flow were evaluated at baseline and 72 hours after IR, hearts were explanted and mitochondrial ROS generation was measured using MitoPY1, as well as protein levels of Thbs‐1, Pgc‐1α, and Drp‐1. In vitro, rat aortic endothelial cells (RAEC) were treated with siRNA or plasmid for Pgc‐1α to evaluate Pgc‐1α effect on Drp‐1.

**Results:**

Mitochondrial ROS generation in heart tissue increased in both age groups following IR. Old animals exhibited diastolic dysfunction at baseline; after IR they displayed reduced systolic function and exacerbated diastolic dysfunction compared to young controls. IR increased Thbs‐1 and Drp‐1 expression in young and old hearts compared to control. siRNA to Pgc‐1α enhanced levels of Drp‐1 in RAECs and increased ROS generation after hypoxia, while Pgc‐1α plasmid ameliorates Drp‐1 expression in the presence of exogenous Thbs‐1.

**Conclusion:**

These results highlight a novel signaling pathway by which Thbs‐1 regulates mitochondrial fission protein (Drp‐1) and ROS generation during hypoxia, and presumably, following IR. Inhibiting Thbs‐1 immediately after IR may prevent Drp‐1‐mediated mitochondrial fission and is likely to improve the diastolic function of the heart by reducing ROS‐mediated cardiomyocyte damage in the aged population.

## 1 INTRODUCTION

The rapid growth of the global elderly population has heightened awareness of age‐related diseases, including interest in the study of the aging heart. Cardiovascular diseases are the leading cause of mortality in the elderly population, as those older than 65 years account for greater than 80% of patients with ischemic heart disease.^1^ The majority of cardiovascular diseases, such as acute myocardial infarction and its correction with percutaneous transluminal coronary angioplasty, known as ischemia‐reperfusion (IR), occur in this aged population.^2^ The growing evidence from both animal experiments and clinical observations indicates that myocardial dysfunction after IR is caused not only by necrosis, a traditional cell death pathway, but also by free radical damage of the myocardial tissue.^3^ Contractile abnormalities in post‐ischemic myocardium were once thought to be the result of irreversible cellular damage and loss of viable myocardium.^3^, ^4^ However, it is now clear that cardiac dysfunction can persist after reperfusion despite restoration of normal or near‐normal coronary blood flow.^3^ Myocardial dysfunction appears to result from reperfusion, which triggers the generation of reactive oxygen species (ROS), an oxygen paradox.^5^, ^6^

Mitochondria are the main source of cellular ROS and contain a number of enzymes that convert molecular oxygen to superoxide (O_2_^−^) or its derivative hydrogen peroxide (H_2_O_2_).^7^ Overproduction of ROS by mitochondria plays a role in the pathogenesis of myocardial IR.^3^ In turn, myocardial IR activates mitochondrial fission marker dynamin‐related protein‐1 (Drp‐1), leading to altered mitochondrial dynamics, increased ROS generation, and left ventricular diastolic dysfunction (LVDD).^8^

Another known contributor of ROS production in aged animals is Thrombospondin 1 (Thbs‐1), which has also been shown to result in deranged vascular reactivity.^9^, ^10^, ^11^ A recent study describes Thbs‐1 as a novel mediator of IR injury by inducing cellular ROS generation in vascular tissue, but the signaling mechanisms have not been identified.^12^ In addition, controlling Drp‐1‐mediated excessive mitochondrial fission after myocardial ischemia has been shown to contribute to the prevention of long‐term cardiac dysfunction.^13^ Loss of signaling from the Thbs‐1 system leads to accumulation of normally functioning mitochondria, stemming from lower ROS production and more efficient metabolism.^14^ Pgc‐1α regulates mitochondrial biogenesis,^15^ and its expression is regulated by cGMP and cAMP signaling, both of which are implicated as important contributors to failing hearts.^14^ Since Thbs‐1 suppresses both cGMP and cAMP levels, and cyclic nucleotides are reported to stimulate mitochondrial biogenesis,^14^ our current hypothesis is that inhibition of Thbs‐1 will reduce Drp‐1 mediated mitochondrial fission through enhancement of Pgc‐1α.

The aim of the present investigation was to establish a signaling link between Thbs‐1 and Drp‐1 through Pgc‐1α following IR in advancing age. Our data showed that IR induces ROS generation and increased Thbs‐1 expression in hearts, potentially leading to PGC‐1‐ and Drp‐1‐mediated mitochondrial fission.

## 2 METHODS

All animal surgeries were performed in accordance with protocols approved by the University of Louisville Institutional Animal Care and Use Committee (IACUC‐approved protocol #18223) and the NIH *Guide for the Care and Use of Laboratory Animals*.^16^

The female Fischer‐344 rat model was selected due to the inbred background of the animals, the absence of large‐vessel CVD as the colony ages, and the development of aging‐induced CMD which resembles the clinical scenario in aging humans.^17^ Young (3 mo) and old (22 mo) female Fischer‐344 rats (Harlan Laboratories and National Institute on Aging, respectively) were housed in groups with free access to food and water and were maintained on a regular 12‐hour light/dark cycle. Young rats were acclimated to facility conditions for a minimum of one week prior to endpoint procedures. Old rats were acclimated to facility conditions for a minimum of one week prior to “baseline” ultrasound scanning. Animals were then divided into four groups, including young control (YC), old control (OC), young IR (Y IR), and old IR (O IR). After 72 hours of IR, all rats were randomly divided into subgroups for endpoint procedures: echocardiography, isolated coronary arteriole experiments, or histology. Animals were deeply anesthetized with 5% isoflurane‐balanced O_2_ before being euthanized by removal of the heart.

### 2.1 Model of IR injury

Ischemia reperfusion injury was induced as previously described.^18^ Briefly, anesthesia was induced with 5% isoflurane at 1 L/minute O_2_ flow followed by maintenance anesthesia of 1.5–2.0% isoflurane with 1 L/minute O2 flow. Depth of anesthesia was confirmed by lack of withdrawal reflex, and ophthalmic vet ointment was applied to the eyes to prevent dryness while under anesthesia. The proper body temperature of 37°C‐38°C was maintained using a heating pad and animal temperature controller. Pre‐surgical Meloxicam, 5 mg/kg, was given intramuscularly 15 minutes before surgery, followed by 2 mL subcutaneous bolus of 0.9% saline to prevent dehydration during the surgery. Rats were intubated and connected to a ventilator for maintenance anesthesia. To induce ischemia, the left anterior descending artery (LAD) was exposed via a 15 mm opening at the 5th intercostal space, then ligated for 30 minutes using 8‐0 monofilament suture. The ligature was released after 30 minutes and reperfusion verified by reddening of the previously discolored area of heart muscle for 1‐2 minutes. The rib cage was closed using 4‐0 absorbable suture with an interrupted suture pattern, and the skin was closed using 5‐0 non‐absorbable silk suture with continuous suture pattern. Rats were removed from anesthesia and were allowed to recover in the home cage.

### 2.2 Measurement of mitochondrial superoxide generation

Mitochondrial superoxide generation was assessed by MitoSOX Red (Molecular Probes) staining, a fluorogenic dye that is taken up by mitochondria, where it is readily oxidized by superoxide. Fresh frozen 5‐μm LV slices were incubated for 20 minutes at 37°C with1.5 μM MitoSOX Red and fluorescence imaged at 60× magnification via an Olympus FluoView 1000 laser scanning confocal microscope (Olympus America). Total mean fluorescence (red) intensity (MFI) in five random fields (for each experiment) was measured with Nikon Elements image analysis software (Nikon Instruments). Fluorescence intensity unit values from LV for each experimental group were averaged and presented as a measure for O_2_^−^ production.

### 2.3 Echocardiography

LV systolic and diastolic function were evaluated by transthoracic echocardiography using a Vevo 3100 with MS250D transducer having a frequency of 13‐24 MHz as previously described 19 (FUJIFILM VisualSonics Inc). Briefly, variables that represent diastolic function—E/A ratio, IVRT, and E/e′—were obtained during resting condition from an apical four‐chamber view with conventional pulsed wave tissue Doppler. E/A ratio was calculated from the peak velocity flow in early diastole (the E wave) to peak velocity flow in late diastole caused by atrial contraction (the A wave) during resting conditions. Results from five cardiac cycles during expiration were averaged together and were used for between‐group and within‐group (time after 72 hours of IR) comparisons. Number of animals in each group: YC n = 6, OC n = 6, Y IR n = 6, and O IR n = 6.

In addition to the standard echocardiographic imaging of cardiac function, a modified parasternal short‐axis projection was used for Doppler recording of the LAD during rest while animals were anesthetized with 1.5% inhaled isoflurane, and again during dobutamine dose (20 g/kg/minute). LAD velocity pre‐dobutamine and during the dobutamine stress challenge were averaged from three consecutive cardiac cycles and CFR was calculated as the ratio of the mean peak LAD velocity values during stress and rest.^18^, ^20^

### 2.4 Immunohistochemistry

Hearts were collected at the endpoint (after 72 hours of IR, and controls after ultrasound data were collected) from experimental animals after KCl injection (40 mM). Hearts were thoroughly washed in PBS. For fixation, hearts were perfused with 4% paraformaldehyde and preserved in Peel‐A‐Way disposable plastic tissue embedding molds (Polysciences Inc) filled with tissue freezing media (Triangle Biomedical Sciences) and stored at −70°C until analysis. Tissue sections (5 µm in thickness) were cut using a Leica CM 3050S Cryostat. Sections were placed on Superfrost plus glass slides, air‐dried, and processed for histological and immunohistochemical (IHC) staining using a standard IHC protocol. Primary antibodies applied overnight included anti‐Drp‐1 and Thbs‐1 (all from Abcam). Secondary antibodies labeled with Alexa Fluor 488 and Texas Red (Invitrogen) were applied for immunodetection of these proteins. Stained slides were imaged for fluorescence at 100× magnification using an Olympus FluoView 1000 laser scanning confocal microscope. The total fluorescence (green or red) intensity in five random fields (for each experimental sample) was measured with Nikon Elements image analysis software. Fluorescence intensity values for each experimental group were averaged and presented as mean fluorescent intensity (MFI).

### 2.5 Fluorescence detection of mitochondrial H_2_O_2_

Hearts were removed from a second subset of animals at the endpoint (after 72 hours of IR, or after ultrasound collection in control animals). Coronary arterioles (approximately 100 µm in diameter) from the LAD artery distribution were isolated and transferred to a vessel chamber (Living Systems, Inc), cannulated on both ends, then pressurized to 30 mm Hg. Mito Peroxy Yellow 1 (Mito PY1, Tocris Bioscience) was used to evaluate mitochondrial‐derived H_2_O_2_ in vessels during flow as previously described.^21^ Following cannulation in a warmed chamber (37°C) containing HEPES buffer (pH 7.4), arterioles were perfused intraluminally with MitoPY1 at 10 µM concentration for 30 minutes. After 30 minutes of incubation, the pressure was increased to 45 mm Hg and incubation in MitoPY1 continued for an additional 30 minutes. Baseline measurements of fluorescence were obtained in the absence of flow (0 µL/minute flow for 2 minutes); images were captured on an Olympus DP80 camera using the TRITC filter and epifluorescent lamp in Olympus CellSens software. The flow was gradually increased up to 5 µL/minute flow for 2 minutes, followed by 25 µL/minute flow for 2 minutes; images were captured after each flow increase. Relative fluorescence intensity (vessel fluorescence minus background fluorescence; arbitrary units) was analyzed with Nikon Elements software.

### 2.6 Cell transfection

Unlabeled rat aortic endothelial cells (RAEC) were obtained at passage 3 from Angio‐Proteomie. RAECs were grown in RAEC culture media (RCM: DMEM, FBS, HEPES, L‐glutamine, ECGS) on 1% gelatin‐coated flasks in 5% CO_2_ incubator. Media was changed every other day. Cells were passaged upon reaching ~80% confluency and split 1:3 until trypsinized at passage 5‐6. The hypoxia model was established using the hypoxia‐inducing agent CoCl_2_ (Sigma‐Aldrich), with a concentration of 100 µM for 24 hours, cell were washed and fresh cell media was added to mimic reperfusion.^22^, ^23^ Knockdown of Pgc‐1α expression was achieved using RNA interference techniques 24 and enhancement of Pgc‐1α was achieved using a pEGFP‐C1 mammalian expression vector containing full‐length Pgc‐1α insert.^25^ Differentiated, serum‐starved RAEC cells were treated with siRNA for either Pgc‐1α (SC‐72151, Santa Cruz Biotechnology) or control scrambled siRNA (SC‐37007). For overexpression, cells were transfected with Pgc‐1α construct, as stated above, for 48 hours (Addgene).^25^ Thbs‐1 was added at a concentration of 2.2 nM for 24 hours post‐transfection (recombinant human, Athens Research and Technology).^9^

### 2.7 Protein extraction and protein estimation

RAEC were sonicated in ice‐cold radioimmunoprecipitation assay buffer (RIPA) (1 mmol/L) with PMSF and protease inhibitor cocktails (1 μL/mL of lysis buffer, Sigma‐Aldrich). The samples were centrifuged at 13 400 g for 30 minutes at 4°C. The supernatant was stored at –80°C until use. Protein estimation was measured by Bradford‐dye (Bio‐Rad) method in 96‐well microtiter plate against bovine serum albumin standard. The plate was analyzed at 594 nm in Spectra Max M2 plate reader (Molecular Devices Corporation).

### 2.8 Western blot analysis

The prepared protein lysate (60 μg) was heated at 95°C for 5 minutes and loaded on sodium dodecyl sulfate polyacrylamide (SDS‐PAGE) gels in running buffer and run at constant current (100 V). Proteins were then transferred to a PVDF membrane overnight at 120 mA. After transfer, the membranes were blocked in 5% nonfat milk for 1 hour at room temperature, followed by overnight incubation with primary antibodies (anti‐Thbs‐1, Drp‐1, and Pgc‐1α; Abcam) at 4°C. After washing with TBS‐T buffer, membranes were incubated with secondary antibodies (horseradish peroxidase‐conjugated goat anti‐mouse, goat anti‐rabbit, and rabbit anti‐goat IgG; Abcam) for 1 hour at room temperature with 1:5000 dilution followed by washing. The membranes were developed with ECL Western blotting detection system (GE Healthcare) and all images were recorded in the gel documentation system ChemiDoc XRS (Bio‐Rad). The membranes were stripped with stripping buffer (Boston BioProducts) followed by blocking step with 5% nonfat milk for 1 hour at room temperature. After washing, membranes were re‐probed with anti‐GAPDH antibody (Millipore) as a loading control protein. The data were analyzed by Bio‐Rad Image Lab densitometry software and normalized to anti‐GAPDH bands.

## 3 RESULTS

### 3.1 IR increases ROS generation in cardiomyocytes

**Figure 1:**
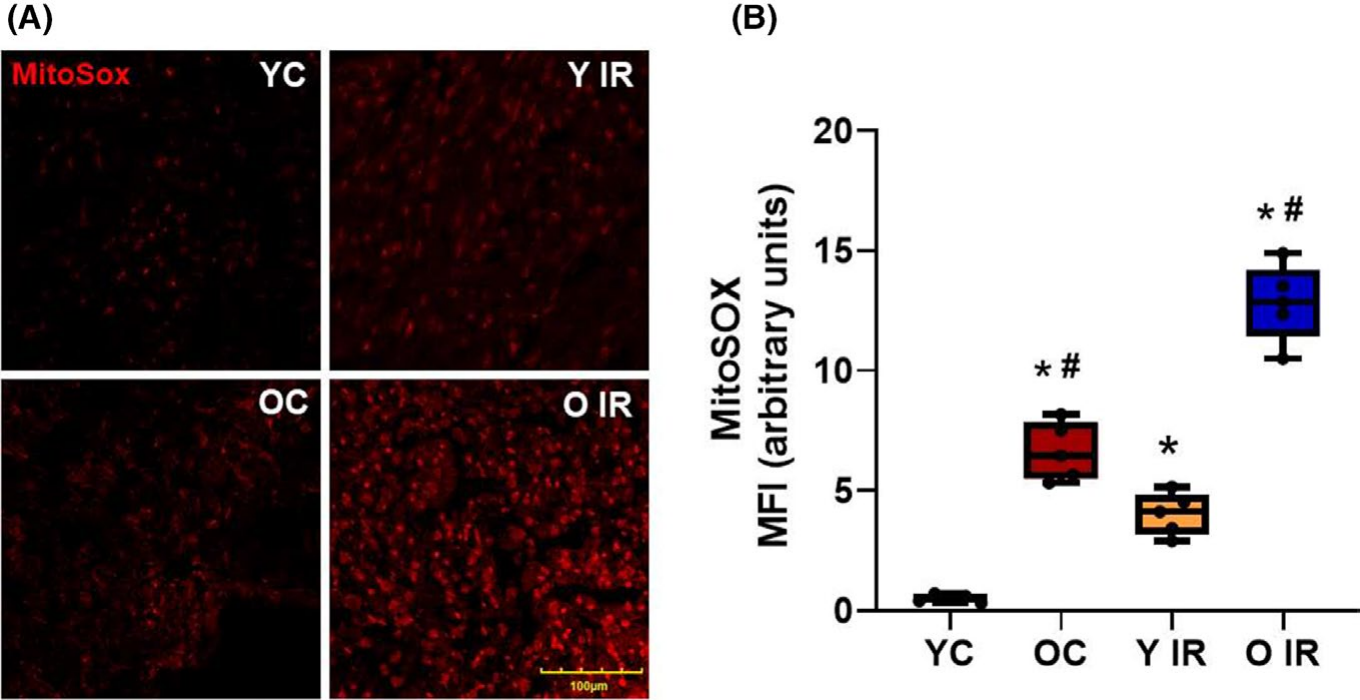
Ischemia reperfusion (IR) induces mitochondrial superoxide generation in heart tissue. Superoxide levels were increased 3 d after IR in both young and old animals. A, Immunofluorescence staining of heart tissue with MitoSOX. B, Mean fluorescence intensity of MitoSOX expression in heart, *P < .05 vs young control, ^#^ *P* < .05 vs old control. YC n = 6, Y IR n = 6, OC n = 6, O IR n = 6. YC, young control; Y IR, young IR; OC, old control; O IR, old IR

To detect the generation of ROS in fresh heart muscle, Mito‐SOX staining was performed on fresh frozen sections of explanted hearts. We observed increased ROS generation in young rats after IR; MFI was increased from 0.488 ± 0.170 to 6.40 ± 1.27, n = 6 (Figure 1). We identified increased ROS generation in hearts from aged animals even without any obvious coronary injury (MFI = 4.31 ± 0.720, n = 6), which was exacerbated after IR (MFI = 13.4 ± 1.10, n = 6) (Figure 1).

### 3.2 IR leads to cardiac dysfunction

**Figure 2:**
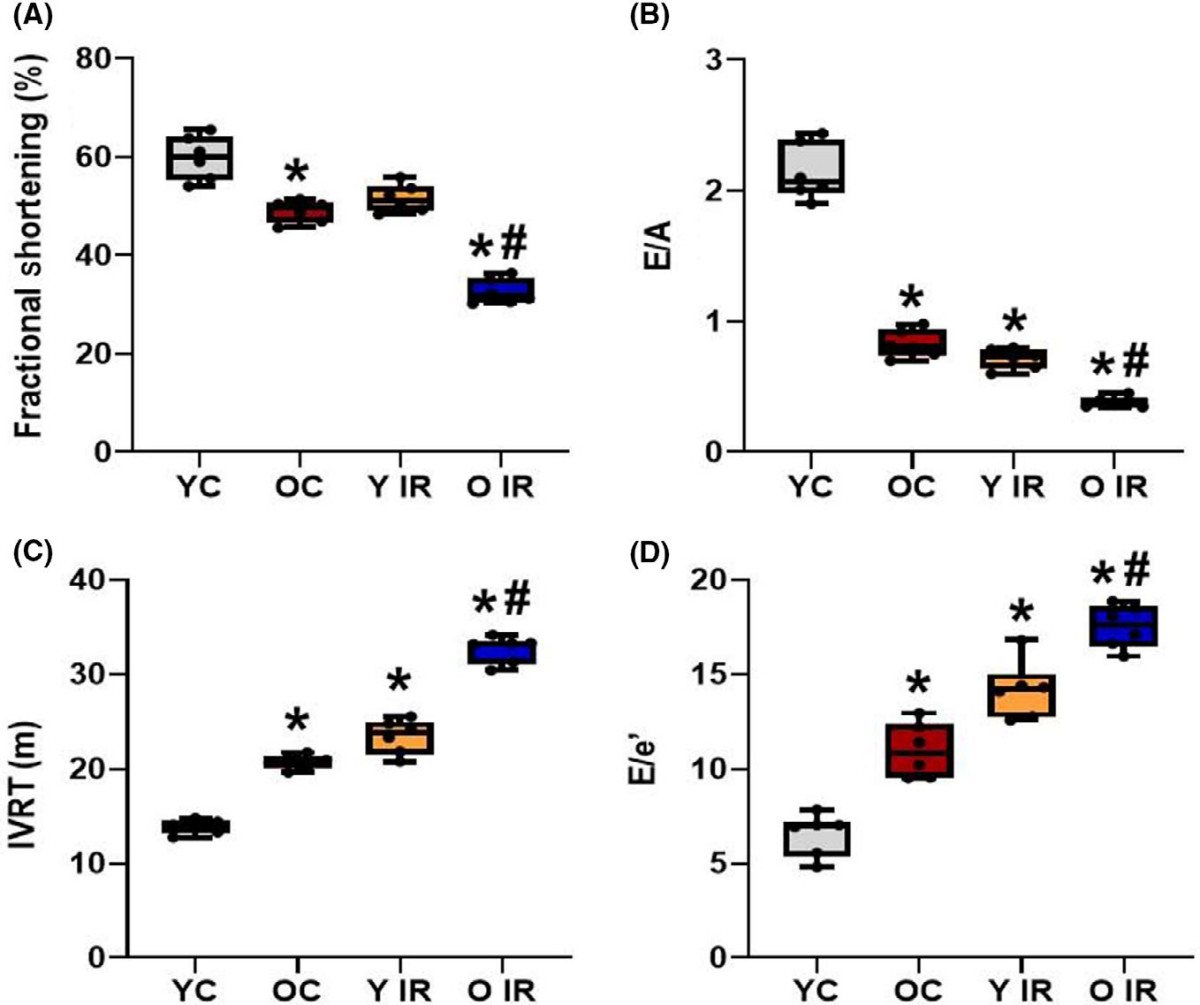
Ischemia reperfusion (IR) reduces systolic function and enhances already existing diastolic dysfunction in old animals. A, Fractional shortening (%) calculations for all experimental groups. B, E/A ratio, C, Isovolumic relaxation time (IVRT), and D, E/e′ calculations from flow velocity of experimental groups, where *P < .05 vs young control, and ^#^ *P* < .05 vs old control. YC n = 6, Y IR n = 6, OC n = 6, O IR n = 6. YC, young control; Y IR, young IR; OC, old control; O IR, old IR

Following IR, there is evidence of cardiac dysfunction, especially LVDD (Figure 2). In older control rats, systolic function was preserved (55.9 ± 1.80%, n = 6) but declined after IR (32.8 ± 3.10%, n = 6) (Figure 2A). LVDD was evaluated by measuring E/A ratio, which decreased following IR in both young (0.829 ± 0.105, n = 6) and old animals (0.380 ± 0.039, n = 6) (Figure 2B). Aging alone also exhibited signs of LVDD by decreasing the E/A ratio (0.717 ± 0.077, n = 6). Isovolumic relaxation time (IVRT) is another diagnostic tool for LVDD. Prolonged IVRT was exhibited in aged animals (23.4 ± 1.82, n = 6) and exacerbated following IR (32.7 ± 1.43, n = 6) (Figure 2C). E/e′ showed a similar trend to IVRT (YC 6.54 ± 1.13, Y IR 11.0 ± 1.44, OC 14.2 ± 1.52, O IR 17.5 ± 1.14, n = 6) (Figure 2D).

**Figure 3:**
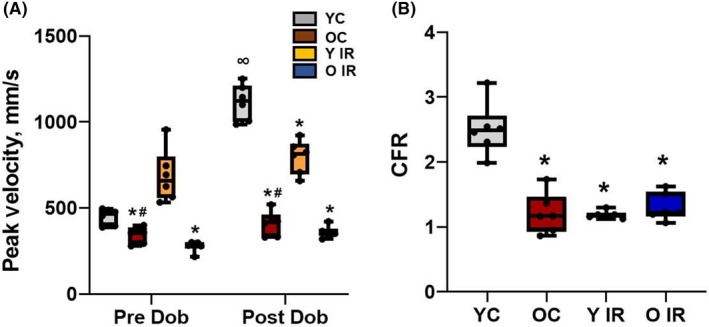
Ischemia reperfusion (IR) affects coronary flow in young animals. A, Coronary peak velocity changes during the dobutamine stress test from experimental groups. B, CFR calculated and presented from experimental groups. *P < .05 vs young control, and ^#^ *P* < .05 vs old control. ^∞^ *P* < .05 post vs pre‐dobutamine, YC n = 6, Y IR n = 6, OC n = 6, O IR n = 6. YC, young control; Y IR, young IR; OC, old control; O IR, old IR

In order to evaluate resting coronary artery function and the response to a cardiac challenge, Doppler recording of the coronary blood flow velocity of the LAD was obtained in resting and dobutamine conditions (Figure 3A). Absolute resting LAD flow velocities showed an increase in young IR animals (YC 450 ± 47.2, Y IR 686 ± 154, n = 6), and was significantly decreased in old animals regardless of IR (OC 344 ± 48.3, O IR 278 ± 31.4, n = 6) (Figure 3A). At the end of dobutamine infusion, the young control group exhibited increased absolute LAD velocity (1114 ± 105 mm/second, n = 6) (Figure 3A) but this response was diminished in young IR (796 ± 97.5 mm/second, n = 6), old control (410 ± 71.8 mm/second, n = 6) and old IR (358 ± 34.8 mm/second, n = 6) (Figure 3A). CFR was assessed because it is a functional measure of large‐ and small‐vessel ischemia and is an indicator of coronary microvascular function. CFR was significantly reduced in the young group after IR and in both aged groups regardless of IR (Figure 3B).

### 3.3 Flow‐Induced mitochondrial H_2_O_2_ generation is enhanced in coronary arterioles from IR subjects

**Figure 4:**
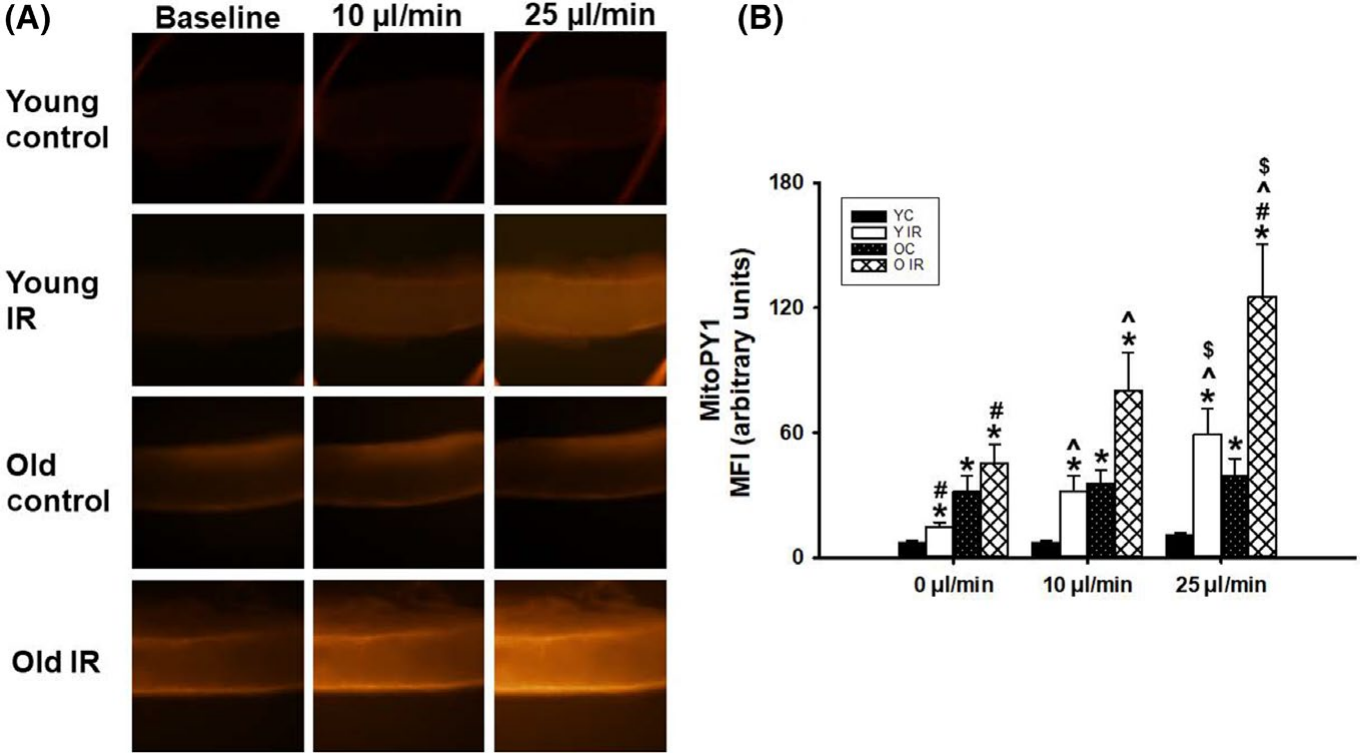
Source of H_2_O_2_ after Ischemia reperfusion (IR) overexpression on flow‐mediated dilation (FMD) in coronary arteries. A, Enhancement in MitoPY1 fluorescence after 72 h of IR. Changes in fluorescence intensity in response to shear stress were evaluated in IR vessels vs control; B, Graphical representation of mitoPY1 overexpression in old and young rats with/without IR. *P < .05 vs young control, ^#^ *P* < .05 vs old control, ^^^ *P* < .05 vs 0 µL/min flow, ^$^ *P* < .05 vs 10 µL/min flow, YC n = 3, Y IR n = 3, OC n = 3, O IR = 3. YC, young control; Y IR, young IR; OC, old control; O IR, old IR

To investigate whether flow‐induced ROS generation increases in occluded coronary arteries following IR, mitochondrial H_2_O_2_ generation was measured via the fluorescent dye MitoPY1. Flow induction did not induce H_2_O_2_ generation in young vessels. However, flow induction in young animals after IR showed a significant increase in MitoPY1 fluorescence compared to baseline (Figure 4). Additionally, an increase in MitoPY1 fluorescence was observed in coronary arterioles from old animals and was enhanced further in coronary arterioles from aged IR animals (Figure 4).

### 3.4 IR enhances Thbs‐1 and Drp‐1 expression in heart tissue

**Figure 5:**
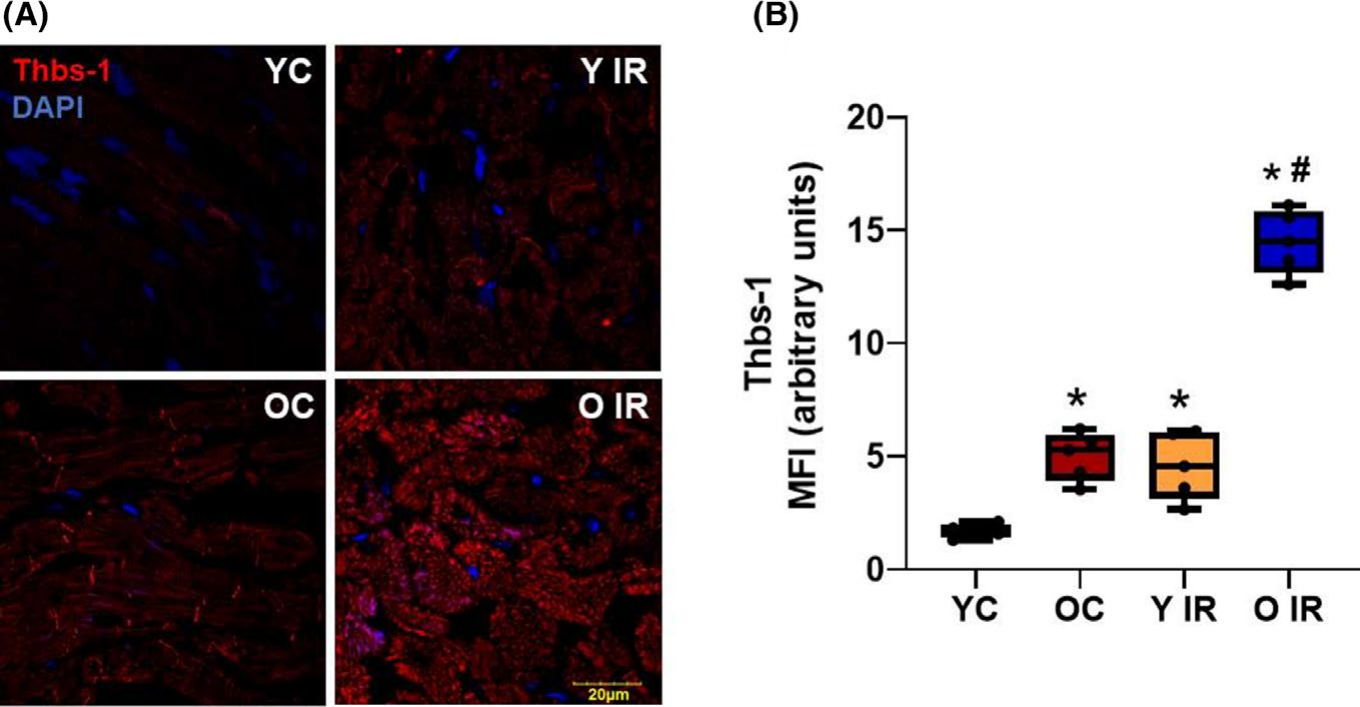
Thbs‐1 expression is increased with advancing age and further exacerbated by Ischemia reperfusion (IR). A, Immunofluorescence staining of heart tissue with Thbs‐1. B, Mean fluorescence intensity of Thbs‐1 expression in heart, *P < .05 vs young control, ^#^ *P* < .05 vs old control. YC n = 6, Y IR n = 6, OC n = 6, O IR n = 6. YC, young control; Y IR, young IR; OC, old control; O IR, old IR

**Figure 6:**
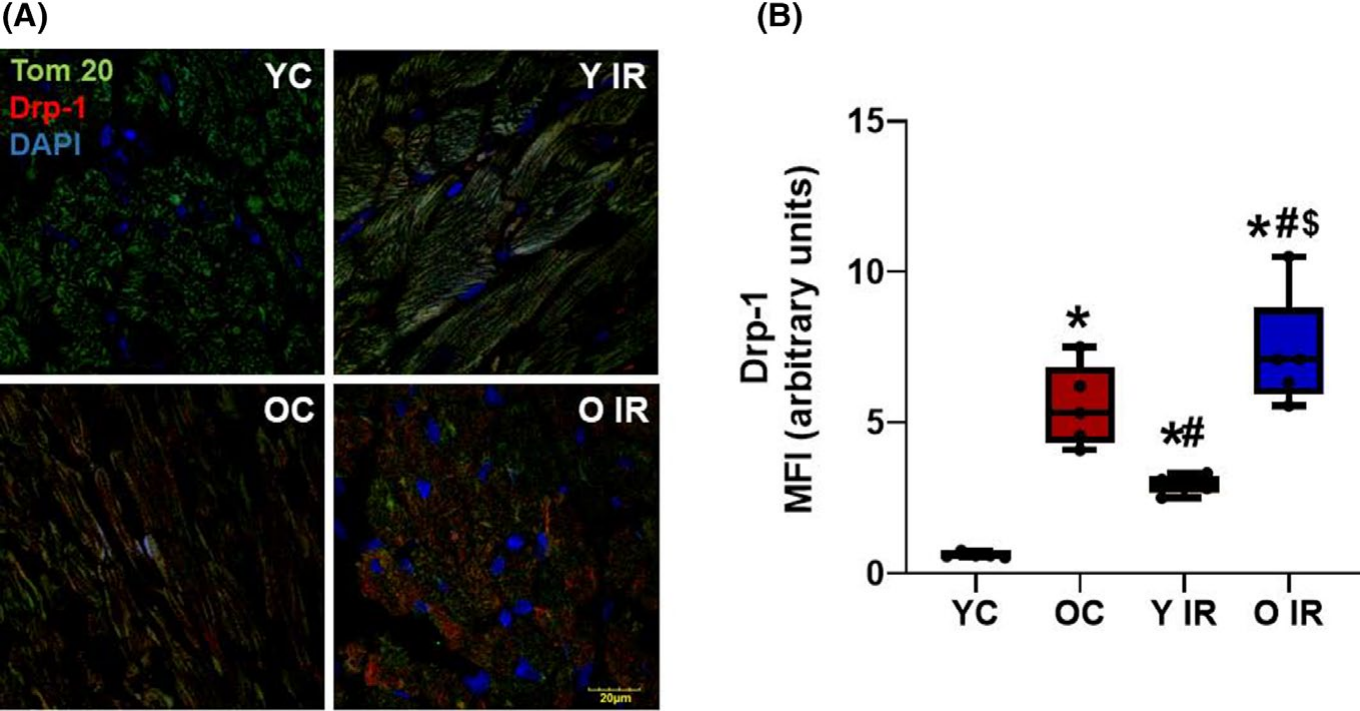
Ischemia reperfusion (IR) induces Drp‐1 expression. Histological analysis of rat heart, representative images of immunofluorescence staining for Drp‐1. A, Immunofluorescence staining of heart tissue with Tom20 (a marker of mitochondria), Drp‐1, and DAPI (nuclear marker). B, Mean fluorescence intensity of Drp‐1 expression in heart, *P < .05 vs young control, ^#^ *P* < .05 vs old control, ^$^ *P* < .05 vs Young IR, YC n = 6, Y IR n = 6, OC n = 6, O IR n = 6. YC, young control; Y IR, young IR; OC, old control; O IR, old IR

To determine whether Thbs‐1 and Drp‐1 expression levels were increased following IR, we measured Thbs‐1 and Drp‐1 expression in heart tissue. We found that IR increased Thbs‐1 and Drp‐1 expression in young animals (Figures 5 and 6). These proteins were increased in aged animals before IR, and further enhanced following IR (Figures 5 and 6).

### 3.5 Thbs‐1 regulates mitochondrial fission through Pgc‐1α pathway

**Figure 7:**
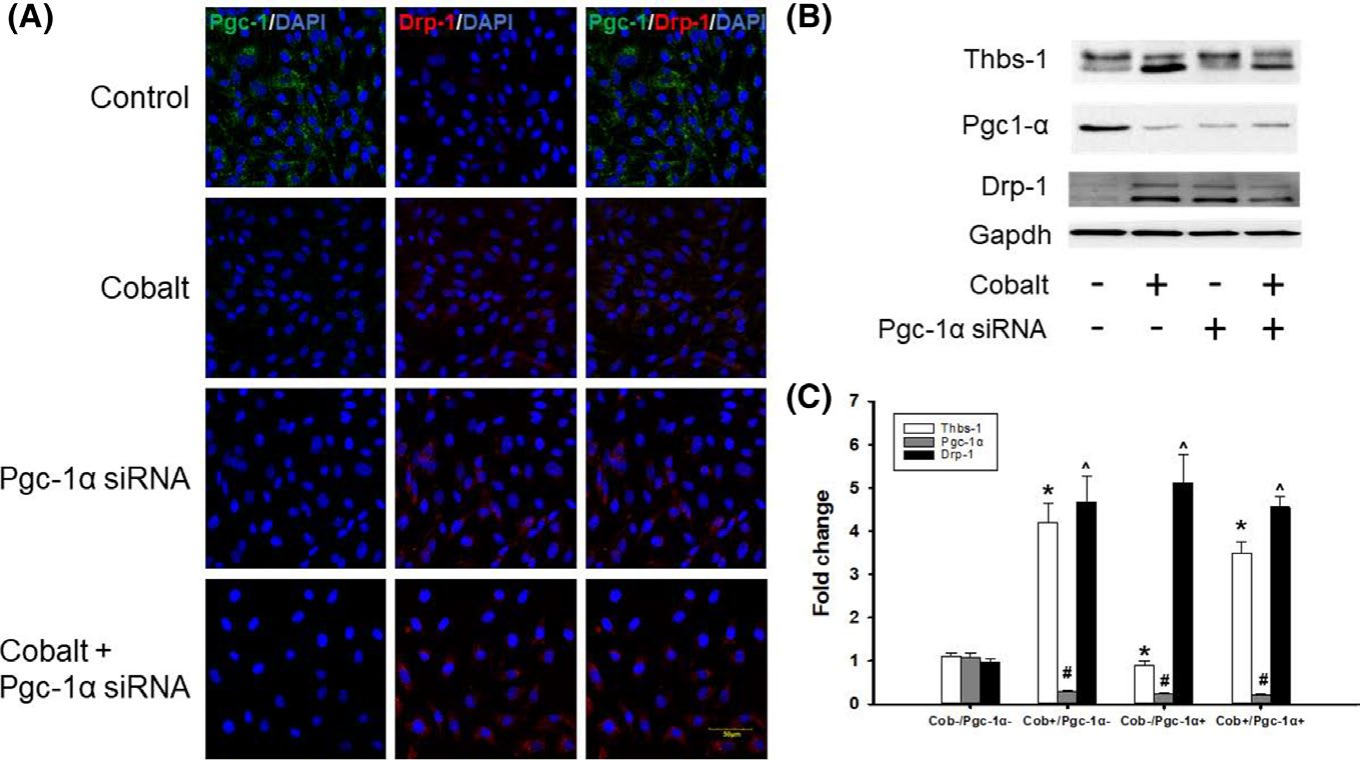
Mitochondrial fission protein Drp‐1 is regulated through Pgc‐1α pathway. A, Fluorescence microscopy images of RAECs with or without exposure to 100 µM CoCl_2_ treated with siRNA for either Pgc‐1α or control scrambled siRNA. B, Western blot analysis was performed using specific antibodies against the indicated proteins after being treated with the indicated siRNA. Blots were reprobed for Gapdh to normalize each lane for protein content; C, Graphical representation of protein expression fold change. n = 5 from each group. *Thbs‐1 treated groups vs Cob ‐/Pgc‐1α, ^#^Pgc‐1α treated groups vs Cob ‐/Pgc‐1α, ^^^Drp‐1 treated groups vs Cob ‐/Pgc‐1α

To investigate how hypoxia contributes to the induction of mitochondrial fission‐mediated Pgc‐1α and Drp‐1 levels, we examined whether cobalt treatment (a hypoxic stimulus) affected Thbs‐1 downstream signaling. In addition, we determined whether Pgc‐1α depletion using siRNA against Pgc‐1α altered this pathway (Figure 7). siRNA of Pgc‐1α in RAEC led to enhanced Drp‐1 expression in the cells. The Thbs‐1 level was enhanced after CoCl_2_ and was not changed regardless of siRNA transfection (Figure 7).

**Figure 8:**
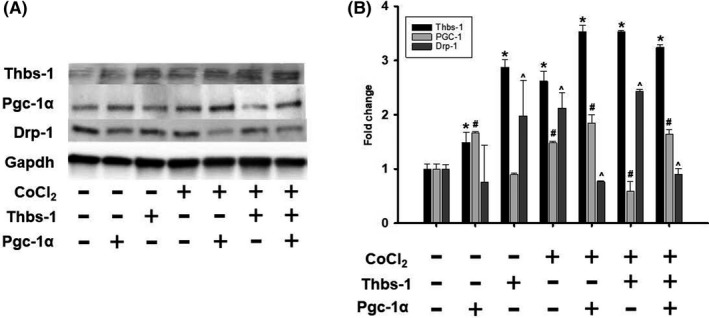
Thbs‐1 is linked to mitochondrial fission protein Drp‐1 and is regulated through Pgc‐1α pathway. A, Western blot analysis was performed using specific antibodies against the indicated proteins after being treated with PGC‐1 plasmid, and exogenous Thbs‐1. Blots were reprobed for GAPDH to normalize each lane for protein content; B, Graphical representation of protein expression fold change. n = 5 from each group. *Thbs‐1 treated groups vs Cob ‐/Pgc‐1α, ^#^Pgc‐1α treated groups vs Cob ‐/Pgc‐1α, ^^^Drp‐1 treated groups vs Cob ‐/Pgc‐1α

To determine how Pgc‐1α contributes to the Thbs‐1 and Drp‐1 signaling pathway, we used Pgc‐1α plasmid to enhance the levels of Pgc‐1α in RAEC with or without hypoxia along with exogenous Thbs‐1 treatment. We found that regardless of hypoxia and exogenous Thbs‐1, Pgc‐1α plasmid enhanced the Pgc‐1α protein expression (Figure 8). Pgc‐1α plasmid decreased Drp‐1 levels, even during hypoxia and Thbs‐1 treatment (Figure 8).

## 4 DISCUSSION

Mitochondria have long been a possible culprit with the onset and progression of age‐associated disease. While the impact of mitochondrial ROS is still under investigation, the declining mitochondrial function seems to be a major contributing factor to the aging process, especially during heart failure. Post‐ischemia cardiac function is significantly decreased in older age, which further augments reperfusion‐induced coronary dysfunction.^6^, ^26^, ^27^ Although aging is associated with an increased generation and presence of ROS in nearly all tissues,^11^ scientists are still debating if aging directly increases ROS generation. Some studies show that the formation of ROS increases during aging 28, 29 while others suggest that ROS production is unchanged.^30^ Kuka et al have shown higher ROS production in heart tissue of 24‐25 month‐old rats compared to heart tissue of 4‐8 month‐old rats.^31^

Our data showed that ROS generation was increased in aged animals before IR, and exacerbated further following IR (Figure 1). Systolic function was preserved in aged control animals despite the increase in ROS generation, while a robust increase of ROS production after IR is associated with impairment of systolic function in these animals (Figure 2A). Although diastolic function was compromised in aged animals before IR, LVDD also occurred in young animals after IR (Figure 2B‐D). This is in agreement with our previous study, where we showed that aging was associated with diastolic but not systolic dysfunction of the heart.^19^

Compared to vascular endothelial cells, cardiac myocytes are more sensitive to IR,^32^ but the coronary microvessels can be mediators of augmented myocardial dysfunction after IR injury.^32^ Non‐invasive quantification of coronary microvascular perfusion is accomplished by assessing CFR, or the maximum increase in blood flow velocity through the coronary arteries above the normal resting velocity.^19^ When oxygen demand in the myocardium is increased, as during a dobutamine challenge, coronary arteries have the ability to reduce vascular resistance which leads to an increase in the volume of blood passing through the coronary vessels. Increases in vascular resistance or failure of resistance vessels' relaxation limit myocardial perfusion.^19^, ^33^ Previously, we have shown that CFR was reduced during aging.^19^ We follow this up in the present study by demonstrating that CFR is also reduced following IR (Figure 3B). This dysfunction of the coronary microcirculation progressively worsens with aging (even in the absence of ischemia), especially in women.^34^ This is an important problem to address because recovery from IR injury and coronary microvascular function are greatly compromised in aged hearts.^4^, ^35^, ^36^ Additionally, a paradoxical increase in baseline coronary flow velocity occurred after IR in young animals (Figure 3A), which can be explained by the metabolic vasodilation effects of mitochondrial H_2_O_2_.^5^ This effect was blunted in old animals because aged coronary vessels are likely overwhelmed with H_2_O_2_.^37^ Our data showed that H_2_O_2_ was increased in young rats after IR, which was exacerbated further in old animals (Figure 4). This increase in H_2_O_2_ can blunt the metabolic vasodilation effect in coronary arteries.^21^ Enhanced H_2_O_2_ levels are known to be mediated by Drp‐1 overexpression,^38^ which supports our data shown above.

Mitochondrial ROS generation plays a major role in coronary microvascular dysfunction.^39^ Importantly, our laboratory has previously described the role of Thbs‐1 mediated coronary microvascular dysfunction in advanced age.^9^ The antiangiogenic effect of Thbs‐1 is known to decrease capillarity,^40^, ^41^ which can be detrimental in an IR model. Our data supported this finding, as we observed increased levels of Thbs‐1 in heart tissue isolated from old animals, which was enhanced further following IR (Figure 5). In addition to increased levels of Thbs‐1, our data showed significantly higher levels of Drp‐1 after IR in young animals (Figure 6). Drp‐1 expression was high in old animals before IR, and further increased after IR (Figure 6). These data allow us to investigate whether Thbs‐1 induces Drp‐1 mediated mitochondrial fission through Pgc‐1α, eliciting ROS generation. It is known that inhibition of Drp‐1 reduces ROS generation in cardiomyocytes and prevents long‐term cardiac dysfunction,^13^ but the mechanism(s) of how Drp‐1 is regulated is still unknown. Pgc‐1α is known as a regulator of both mitochondrial biogenesis and mitochondrial fusion/fission dynamics.^42^ Our data showed that the hypoxia‐inducing agent CoCl_2_ reduced levels of Pgc‐1α, while Drp‐1 was increased (Figure 7). In addition, Pgc‐1α siRNA eliminated protein levels of Pgc‐1α in treated cells and increased levels of Drp‐1 expression (Figure 7). As expected, CoCl_2_ increased Thbs‐1 levels in cells, and Thbs‐1 level was not changed following Pgc‐1α siRNA (Figure 7). The novelty of our study is that we show a connection between Thbs‐1 and Drp‐1 though Pgc‐1α (Figure 8). From these data, we can conclude that hypoxia increases Thbs‐1 levels in cells, and mitochondrial fission is regulated through Pgc‐1α.

The primary outcome of this study is a new link between Thbs‐1‐ Pgc1α‐ Drp‐1‐mediated cardiac dysfunction after IR. Thbs‐1 can be considered a new mediator of Drp‐1‐mediated mitochondrial fission through Pgc1α during hypoxic conditions and possibly following IR. This finding gives us an opportunity to use Thbs‐1 inhibitors during and/or after IR to protect the heart from failure. Since any clinically administered therapy is likely to occur during the post‐ischemic phase, the findings presented here offer a new translational advantage. While inhibition of Drp‐1 prior to ischemia has been shown to be cardioprotective,^8^ it still remains under investigation whether Drp‐1 inhibition following ischemia and during reperfusion in vivo would have a similar effect. Drp‐1 is an encouraging therapeutic target for cardiac dysfunction after IR injury during aging. Inhibition of Drp‐1 not only will improve cardiac function after IR, but also will preserve mitochondrial function which will eliminate cardiomyocyte death.

In conclusion, our study has shown that IR induces ROS generation and increases Thbs‐1 expression in hearts; in vitro experiments suggest a link between Thbs‐1 and Drp‐1‐mediated mitochondrial fission during hypoxic conditions that is regulated by the Pgc‐1α pathway. Further study is required to elucidate the mechanistic pathway between these proteins in vivo.

### 4.1 Study limitations

The dilation with dobutamine is partially direct via beta adrenergic receptor activation, but primary indirect due to the simultaneous increase in cardiac metabolism leading to metabolic dilation. We have previously utilized dobutamine‐induced cardiac challenge to elucidate CFR differences in young vs old animals.^19^, ^43^ The patterns of CFR function in young vs old presented in the current manuscript are similar to those previously published.^19^, ^43^ Additionally, Rowe et al 43 have utilized adenosine infusion and dobutamine infusion in the same set of animals to examine whether CFR measurements differ when the cardiac challenge is induced via adenosine receptor activation rather than beta adrenergic receptor activation; a similar pattern of age‐related dysfunction in CFR was found regardless of activator. In addition, CFR can be affected by edema of myocardium after IR. In general, myocardial edema is measured using T2‐weighted cardiac magnetic resonance (CMR). In this study, we did not have access to CMR technology; rather, we measured left ventricular thickness before and after IR using ultrasound and did not find any differences (data not included in manuscript). Carrick et al, have shown bimodal myocardial edema using CMR after reperfusion, which depends on time of ischemia and hemorrhagic level in myocardium.^44^ In our study, because we have 30 minutes of ischemia and 72 hours of reperfusion, we may have one of the following three situations: (a) 30 minutes was not enough to cause osmotic cell swelling, (b) 72 hours was enough to clear this edema in cardiomyocytes, or (c) we cannot observe edema using ultrasound.

The other limitation of this study is to recreate a sufficient in vitro IR model. Limiting nutrients using 2‐dexuglucose on top of hypoxia (induced via CoCl2) might have better simulated the in vivo ischemic stimulus. However, the current experiments only utilized CoCl2 followed by wash out. This being said, other studies 23, 24 have shown that a similar concentration of CoCl2 used in the present study sufficiently induced a hypoxic stimulus.

## CONFLICT OF INTEREST

The authors declare that they have no conflicts of interest.

## AUTHOR CONTRIBUTIONS

Natia Q. Kelm designed the research; Natia Q. Kelm analyzed the data; Natia Q. Kelm, Jason E.Beare, Gregory J. Webber performed the research; Natia Q.Kelm, Jason E. Beare, Gregory J. Weber, Amanda J. LeBlanc wrote the paper.
